# Evidence for impaired glucose metabolism in the striatum, obtained postmortem, from some subjects with schizophrenia

**DOI:** 10.1038/tp.2016.226

**Published:** 2016-11-15

**Authors:** B Dean, N Thomas, E Scarr, M Udawela

**Affiliations:** 1 The Molecular Psychiatry Laboratory, The Florey Institute for Neuroscience and Mental Health, Parkville, VIC, Australia; 2 The Department of Psychiatry, The University of Melbourne, Parkville, VIC, Australia

## Abstract

Studies using central nervous system tissue obtained postmortem suggest pathways involved in energy and metabolism contribute to the pathophysiology of schizophrenia; neuroimaging studies suggesting glucose metabolism is particularly affected in the striatum. To gain information on the status of pathways involved in glucose metabolism in the striatum, we measured levels of glucose, pyruvate, acetyl-CoA and lactate as well as the β subunit of pyruvate dehydrogenase, a rate limiting enzyme, in the postmortem tissue from subjects with schizophrenia and age/sex-matched controls. The subjects with schizophrenia were made up of two subgroups, which could be divided because they either had (muscarinic receptor deficit schizophrenia (MRDS)), or did not have (non-MRDS), a marked deficit in cortical muscarinic receptors. Compared to controls, levels of β subunit of pyruvate dehydrogenase were lower (Δ mean=−20%) and levels of pyruvate (Δ mean=+47%) and lactate (Δ mean=+15%) were significantly higher in the striatum from subjects with schizophrenia. Notably, in subjects with non-MRDS, striatal levels of β subunit of pyruvate dehydrogenase were lower (Δ mean=−29%), whereas levels of pyruvate (Δ mean=−66%), acetyl-CoA (Δ mean=−28%) and glucose (Δ mean=-27%) were higher, whereas levels of lactate (Δ mean=+17%) were higher in MRDS. Finally, discriminate analyses using levels the β subunit of pyruvate dehydrogenase and glucose, or better still, β subunit of pyruvate dehydrogenase and glucose in combination with pyruvate, lactate or acetyl-CoA could separate subjects with non-MRDS from controls with high levels of specificity (up to 93%) and selectivity (up to 91%). Our data show the benefit of being able to study defined subgroups within the syndrome of schizophrenia as such an approach has revealed that changes in glucose metabolism may be a significant contributor to the pathophysiology of non-MRDS.

## Introduction

Studies of gene expression in human central nervous system (CNS) suggest that dysregulated metabolic pathways contribute the pathophysiology of the schizophrenia.^1^ The notion that changes in metabolic pathways are disturbed in subjects with schizophrenia has gained further supported from studies of the human proteome^2^ and metabolome^3^ in CNS tissue from subjects with schizophrenia. Significantly, studies on the human CNS metabolome broadly implicate changes in glucose metabolism in the pathophysiology of schizophrenia.^3^ This is important because the CNS does not have extensive energy reserves in the form of glycogen and therefore relies heavily on producing its energy requirements from available glucose.^4^ Thus, any change in glucose metabolism could affect nearly all cellular process in neurons and glia, thus impacting on processes including neurotransmission and rates of cell death. Moreover, changes in glucose metabolism in the CNS could particularly impact neuronal function because, on a per cell basis, neurons make the highest energy demand and therefore require ready access to glucose for metabolism.^5^ As in any cell, neurons metabolized glucose through a number of steps, the end product of which is the generation of cellular energy in a number of forms including ATP, which can also act as a neurotransmitter in its own right.^4^ Thus, any significant changes in glucose metabolism in neurons, in particular, would have profound effect on CNS function which could lead to disorders such as schizophrenia.

Two pathways that have a major role in glucose metabolism are the Embden–Meyerhof pathway and the tricarboxylic acid cycle (TCA).^6^ The Embden–Meyerhof pathway metabolize glucose to pyruvate with a net production of two ATP molecules and two molecules of reduced nicotinamide adenine dinucleotide (NADH), both of which represent energy currency for a cell. Pyruvate can then be converted to acetyl-CoA by the pyruvate dehydrogenase complex with acetyl-CoA being a substrate for the TCA cycle. When one molecule of substrate, acetyl-CoA, is processed through the TCA there is a release of electrons from NADH and reduced flavin adenine dinucleotide (FADH_2_) to facilitate the production of 4 molecules of ATP. It has been reported that the activity of aconitase, α-ketoglutarate dehydrogenase complex and succinate thiokinase is lower in the dorsolateral prefrontal cortex from subjects with schizophrenia.^7^ These data are significant because these enzymes catalyse the first three reactions of the TCA.^6^ In addition, a gene expression array study has reported lower levels of mRNA for succinate dehydrogenase complex subunit A and lactate dehydrogenase A in the hippocampus from subjects with schizophrenia.^8^ Succinate dehydrogenase is another enzyme in the TCA cycle, whereas lactate dehydrogenase can convert pyruvate to lactate, a product that does not enter the Embden–Meyerhof pathway.^6^ Hence, these data suggest that changes in glucose metabolism in the CNS from subjects with schizophrenia may be widespread across a number of regions.

Neuroimaging studies have been used to assess glucose metabolism across the human CNS and such studies have reported changes in glucose utilization in the CNS of subjects with schizophrenia.^9^ Significantly, changes in glucose utilization in the striatum in subjects with schizophrenia have been reported in a number of neuroimaging studies.^10, 11, 12, 13, 14^ These data suggest the striatum may be particularly affected by changes in glucose metabolism in subjects with schizophrenia. Despite such neuroimaging data, postmortem studies do not seem to have focussed on striatum to discover the mechanisms that might underlie changes in glucose metabolism in subjects with schizophrenia.

**Figure 1:**
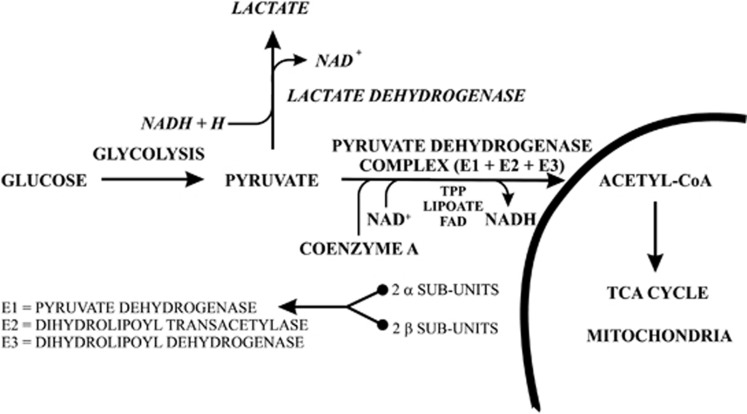
Schematic diagram showing critical components glycolytic pathways controlling the conversion of glucose to acetyl-CoA or lactate. FAD, flavin adenine dinucleotide; NAD, nicotinamide adenine dinucleotide; TPP, thiamine pyrophosphate.

One approach to defining the mechanisms by which glucose metabolism is altered in the CNS from subjects with schizophrenia can be by measuring the activity, protein levels or levels of expression of enzymes in pathways such as the Embden–Meyerhof pathway or the TCA. However, another approach is to measure levels of individual metabolites as surrogate markers of changes in the activity of glucose utilizing metabolic pathways. Using this latter approach, lower levels of lactate have been reported in the anterior limb of the internal capsule from subjects with schizophrenia,^15^ but higher levels of that metabolite were measured in the cerebellum^16^ and cortex^2^ from subjects with the disorder. In addition, compared with controls, lower levels of pyruvate have been reported in the mediodorsal thalamus from subjects with schizophrenia.^17^ In aerobic conditions, which likely dominate most of the time in the CNS, lower levels of pyruvate could be indicative of an increase in processing of this substrate by the pyruvate dehydrogenase complex or a reduced production of pyruvate by the Embden–Meyerhof pathway. By contrast, the conversion of pyruvate to lactate and lactic assay is mostly associated with anaerobic metabolism, a process that is known to begin to dominate sometime after death.^16^ To differentiate between these processes it is important to note that pyruvate dehydrogenase complex is the rate limiting step in the conversion of pyruvate to acetyl-CoA; hence, it would be expected that changes involving aerobic metabolism would be apparent because of changes in the activity or levels of the pyruvate dehydrogenase complex that would be accompanied by inverse changes in the enzymes substrate (pyruvate) and its product (acetyl-CoA) without changes in the anaerobic product (lactate; Figure 1).

The pyruvate dehydrogenase complex is made up of three enzymes, pyruvate dehydrogenase, dihydrolipoyl transacetylase and dihydrolipoyl dehydrogenase.^18^ Pyruvate dehydrogenase is a quadripartite complex made up of 2 α and 2 β subunits with each subunit having roles in regulating the activity of pyruvate dehydrogenase and the pyruvate dehydrogenase complex. Any change in the levels of either of the subunits of the pyruvate dehydrogenase complex would be expected to change the activity of the pyruvate dehydrogenase complex and hence levels of pyruvate and acetyl-CoA. Therefore, the report of changes in levels of pyruvate^17^ in the CNS of subjects with schizophrenia would be consistent with changes in the metabolism of pyruvate by the pyruvate dehydrogenase complex.

The growing body of data from postmortem CNS studies, plus the data from neuroimaging studies and our expression microarray study,^19^ underpinned our decision to measure levels of the β subunits of pyruvate dehydrogenase (PDHB) in the striatum of subjects with schizophrenia and age/sex-matched controls. In addition, to gain further insight into the extent of perturbation of glucose metabolism in the striatum subjects with schizophrenia we measured levels of glucose, acetyl-CoA, lactate and pyruvate (Figure 1). We chose to focus our studies in the striatum because a number of neuroimaging studies have reported changes in glucose utilization in that CNS region in subjects with schizophrenia.^10, 11, 12, 13, 14^

It is now recognized that the diagnoses of schizophrenia defines a syndrome of disorders and that studying the syndrome as a whole is impeding the identification of the core pathophysiologies of the disorders within the syndrome.^20^ We have shown that it is possible to distinguish a subset of individuals within the disorder based on a marked loss of cortical muscarinic receptor binding; we have termed this subset as muscarinic receptor deficit schizophrenia (MRDS). Subjects with MRDS have been shown to have a widespread loss of cortical muscarinic receptors^21, 22^ similar to what was reported in some individuals with the disorder in a neuroimaging study of muscarinic receptors in schizophrenia.^23^ Recently we have reported that subjects with MRDS also have decreased radioligand binding to striatal muscarinic receptors and higher levels of post-synaptic density protein 95,^24^ showing changes in the molecular cytoarchitecture of the CNS in subjects with MRDS extends beyond the cortex. It has long been recognized that muscarinic receptors have been shown to act to modulate glucose uptake in the CNS^25^ and that components of the glucose metabolism pathways, such as PDH, can modulate levels of muscarinic receptors.^26^ Hence, given these recognized interactions between muscarinic receptors and energy and metabolism, we decided to measure levels of markers related to glucose metabolism in the striatum from subjects with schizophrenia and age- and sex-matched controls, with the schizophrenia cohort consisting of subjects with MRDS and subjects with no apparent loss of cortical muscarinic receptors (non-MRDS).

## Materials and methods

### Tissue collection

CNS was initially collected from subjects with a potential history of schizophrenia and subjects with no apparent history of psychiatric disorders who were matched for age and sex. CNS was only collected from people who had been seen alive up to 5 h prior to being found dead and where cadavers had been refrigerated within 5 h to ensure slowing of any autolysis of the CNS.^27^ A postmortem assessment was completed by reviewing histories, discussions with relatives and treating clinicians which, for non-psychiatric controls, was to exclude any history of significant psychiatric symptoms. For each subject with history of schizophrenia relevant clinical and neuropsychopharmacological data were recorded. Subsequently, duration of illness (DI) was calculated as the time from first presentation to a psychiatric service until death and postmortem interval (PMI) was calculated as the time between witnessed death and autopsy or the midpoint between the subject being found and being last seen alive and autopsy. The final recorded dose of antipsychotic drug (FRADD) was noted and converted to chlorpromazine equivalents using algorithms as proposed in the literature,^28^ as was total lifetime exposure to such drugs (LEAP). The diagnosis of schizophrenia was agreed by consensus between two senior psychiatrists and the person completing the assessment according to DSM IV criteria using the Diagnostic Instrument for Brain Studies.^29^

To ensure optimum tissue preservation each left CNS hemisphere was processed in a standardized manner to ensure the tissue was frozen to −70 °C within 30 min of autopsy.^30^ For each case, CNS pH was measured as an indicator of the quality of tissue preservation^31^ as this measure is recognized as giving a good indication as to the biochemical integrity of tissue, which is often not related simply to PMI.^32^ For this study, striatum was cut from frozen slices of tissue from subjects with schizophrenia and age- and sex-matched controls.

In this study, we divide subjects with schizophrenia into two subgroups based on levels of [^3^H]pirenzepine binding in Brodmann's area 9.^33^ Thus, subjects with MRDS have levels of [^3^H]pirenzepine binding in BA 9 of <90 fmol mg^−1^ estimated tissue equivalents whilst non-MRDS have levels of >120 fmol mg^−1^ estimated tissue equivalents.

### Measurement of PDHB

Measurement of PDHB was by western blotting based on established methodologies involving an internal control (IC) but optimized using an anti-human PDHB antibody (Abcam, Melbourne, VIC, Australia; Cat# ab55574). Thus, striatum was homogenized into 10 mM Tris HCl (pH 7.4) containing 1% SDS and 1 mM sodium vanadate on ice using a Potter–Elvehjem grinding chamber and Teflon-coated pestle. The protein concentration of each homogenate was measured prior to the sample being frozen at—70 °C until required. On the day of assay, homogenates were diluted in 0.5 M Tris HCl (pH 6.8) containing 40% SDS, 20% glycerol, 10% β-mecaptoethanol and 0.05% bromophenol blue to give a final 5 μg of protein to be loaded onto a 12% polyacrylamide mini-gel. Following resolving the proteins across the PAGE gel, proteins were transferred to a nitrocellulose membrane.

To measure the intensity of PDHB, which appeared as a 39.1 kDa immunogenic band, the nitrocellulose membrane containing the transferred proteins was blocked with 5% non-fat milk powder in Tris-buffered saline containing 0.1% Tween 20 (0.1% TBST). The blocked membranes were then exposed to 1/100 dilution of an anti-human PDHB antibody (Abcam, Cambridge, UK) for 1hr at r.t. on a rocking platform. Membranes were then washed with 0.1% TBST before being exposed to a goat anti-mouse IgG horse radish peroxidase conjugated antibody (DAKO, North Sydney, NSW, Australia; 1/2000 dilution; P0448) in 0.1% TBST for 1 h at r.t. on a rocking platform. Membranes were again washed with 0.1% TBST and then exposed to Supersignal West Pico Chemiluminescent Substrate (Termo Fisher, Scoresby, Australia) for 5 min at r.t. on a rocker. Excess solution was then drained and blotted off each membrane and the intensity of the chemiluminesence of the PDHB band measure using a Kodak Image Station 440CF.

It is clear that the notion of a reference gene, which is a gene whose expression does not vary during neurodevelopment, between CNS regions or between disorders of the CNS, is not tenable for studies of human CNS at the level of mRNA^34^ or protein.^35^ As suggested,^35^ we have taken an approach to minimize the impact of variation in tissue processing as well as separating and measuring protein levels using Western blotting that does not use a reference protein as a loading control. Hence, de-identified tissue is allocated to the experimenter in batches that contain tissue from subjects with schizophrenia and their matched control. The experimenter processes each batch of tissue so that each Western blot contains an equal number of controls and subjects with schizophrenia; this process ensures the experimenter remains blind to diagnoses. The protein level in each sample is measured for a second time on the morning they are loaded onto a gel to ensure the protein levels loaded onto each gel are accurate. Great care is taken on loading each case, in duplicate, onto the loading gel and following protein transfer, the integrated level of the intensity of Ponceau stained protein bands in each lane is measured to ensure the amount of protein transferred is constant. Finally, to further control for any gel to gel variation an IC homogenate is prepared so that it can be run in every lane of two gels to establish both intra- and interblot variation for measuring the level of the protein of interest in the IC.^36^ The IC is run (in duplicate) on each subsequent gel containing the cases. These gels are exposed so that the sum intensity of the IC falls within the range established as the mean±2 s.d. of the intensities of the two master gels measurements. Finally, the results from all subjects were standardized by expressing them as a ratio of IC. Using this approach we have shown intra- and intergel variation is <10 and <15%, respectively, for measuring any protein of interest.

### Measurement of metabolites

All experimental measures were made in a way that ensured each experimenter was blind to diagnoses.

To measure pyruvate and lactate, striatal tissue was dissected and its wet weight recorded. [^14^C]lactate was added to each tissue sample to give a final concentration of 1.0 nm prior to the tissue being homogenized, on ice, into 540 μl of 0.6% perchloric acid in a Potter–Elvehjem grinding chamber using a Teflon-coated pestle.^37^ Each homogenate was centrifuged at 20 000 *g* for 30 min at 4 °C, the supernatant was collected and adjusted to pH 7.0 with 1.5 m potassium carbonate. The neutralized homogenates were centrifuged at 20 000 *g* for 30 min at 4 °C, the supernatant collected and the level of radioactivity in 20 μl of supernatant measured. The measured radioactivity was divided by the total radioactivity added to the tube before extraction to calculate the extraction efficiency for each sample. Homogenates were frozen at −70 °C until required.

To measure acetyl-CoA and glucose tissue was extracted according to the manufacture's instructions and hence, 40 μl [^3^H]-acetyl-coA was added to 5 ml sample buffer containing 10% PCA. Approximately 100 mg of striatum was then homogenized, on ice, into 5 × volume of sample buffer containing the [^3^H]-acetyl-coA in a Potter–Elvehjem grinding chamber using a Teflon-coated pestle. The homogenate was centrifuged at 12 500 *g* for 10 min at 4 °C and the supernatant was collected and adjusted to pH 6–8 using 3 m K_2_CO_3_. After pH adjustment the homogenate was centrifuged at 12 500 *g* for 2 min at 4 °C. The radioactivity in 3 × 20 μl aliquots of homogenate was counted and the measured radioactivity/total radioactivity added prior to extraction were calculated as the extraction efficiency and each homogenate. Each homogenate was stored at −80 °C until required.

Levels of pyruvate (Abnova, Taipei City, Taiwan), lactate (Sigma-Aldrich, Castle Hill, NSW, Australia), acetyl-CoA (Sigma-Aldrich) and glucose (Arbor Assays, Ann Arbor, MI, USA) were measured using commercially available kits using their recommended protocols. The measurement of pyruvate involved the oxidization of pyruvate by pyruvate oxidase, which causes a change in assay reagent color that can be measured at OD 570 nm. Levels of lactate were measure by a prepared enzymatic assay, which again caused a change in a reagent that could be measured at 570 nm. Acetyl-CoA was measured using a highly sensitive assay in which levels of the metabolite can be determined by a coupled enzyme assay reaction that results in a fluorometric read out (lex=535/lem=587 nm). Finally, glucose was measured by mixing samples with a Colorimetric Substrate containing horse radish peroxidase with the reaction being initiated by addition of glucose oxidase. This reaction produces hydrogen peroxide which, in the presence of horse radish peroxidase, reacts with the Colorimetric Substrate to convert the colorless substrate into a pink-colored product that can be read at 560 nm. Increasing levels of glucose cause a linear increase in color. All measurements for each metabolite were carried out using triplicate samples. Each measurement of pyruvate, lactate, acetyl-CoA and glucose was correct by multiplying by the relevant extraction efficiency. For all methodologies, interassay variation was <3% and intra-assay variation <10%.

### Statistics

The D'Agostino & Pearson omnibus normality test was used to analyze data distribution as this is the best approach when data sets are small.^38^ Unpaired student *t*-tests and one-way analysis of variance with *post hoc* analyses using Dunnett's multiple comparisons test to identify the source of any variance were used to compare numeric demographic, tissue collection and treatment data between the diagnostic cohorts. Non-numeric data relating to gender and suicide completion were compared using *χ*^2^-tests. Levels of PDHB and acetyl-CoA in the striatum from subjects with schizophrenia and age/sex-matched controls were normally distributed and therefore compared using an unpaired student *t*-test, levels of the remaining analytes were compared using the Mann–Whitney test. Levels of PDHB, acetyl-CoA and glucose in striatum from subjects with MRDS, non-MRDS and controls were normally distributed and were therefore compared using one-way analysis of variance with *post hoc* analyses using Dunnett's multiple comparisons test to identify the source of any variance. Levels of pyruvate and lactate across the three diagnostic groups were compared using Kruskal–Wallis and *post hoc* analyses using Dunnett's multiple comparisons test.

Relationships between age, PMI, DI, CNS pH, FRADD and LEAP and experimental data were determined using appropriate linear regression and the resulting coefficient of determination. The small sample sizes in this study meant that only strong relationships, *r*^2^⩾0.49; *ρ<*0.05, warranted further consideration as potential confound. Where there were strong correlations non-experimental data were included as covariates when analyzing experimental data. Finally, discriminate analyses were used to determine the power of metabolic measurements to separate MRDS, non-MRDS and controls with sensitivity and specificity of the analyses determined as described previously.^39^ In these analyses, diagnosis was used to define the groups, whereas the experimental measures were used as the predictors. The discrimination function was linear and no cross-validation was performed.

Statistical analyses were completed using GraphPad Prism (GraphPad Software, La Jolla, CA, USA) and Minitab 15 (Minitab, Sydney, NSW, Australia).

## Results

### Study cohorts

Initial study design was to measure markers of glucose metabolism in the striatum from 39 subjects with schizophrenia and 20 controls based on tissue availability; the cohort from subjects with schizophrenia containing 20 subjects with MRDS. As tissue was used for sequential study insufficient tissue was available for all measurements in all individuals and hence cohort sizes varied between studies (Supplementary Table 1; full details Supplementary Table 2).

### Pyruvate dehydrogenase-β

PDHB was measured in the striatum from 39 subjects with schizophrenia that included 20 subjects with MRDS and 20 age- and sex-matched controls. The were no significant differences in gender frequency, age, CNS pH, PMI or brain weight between the subjects with schizophrenia and the controls (Supplementary Table 1A); similarly, there was no difference in gender frequency, age, CNS pH, PMI or brain weight between subjects with MRDS, non-MRDS and controls or between rates of suicide completion, DI, FRADD and LEAD between MRDS and non-MRDS.

**Figure 2:**
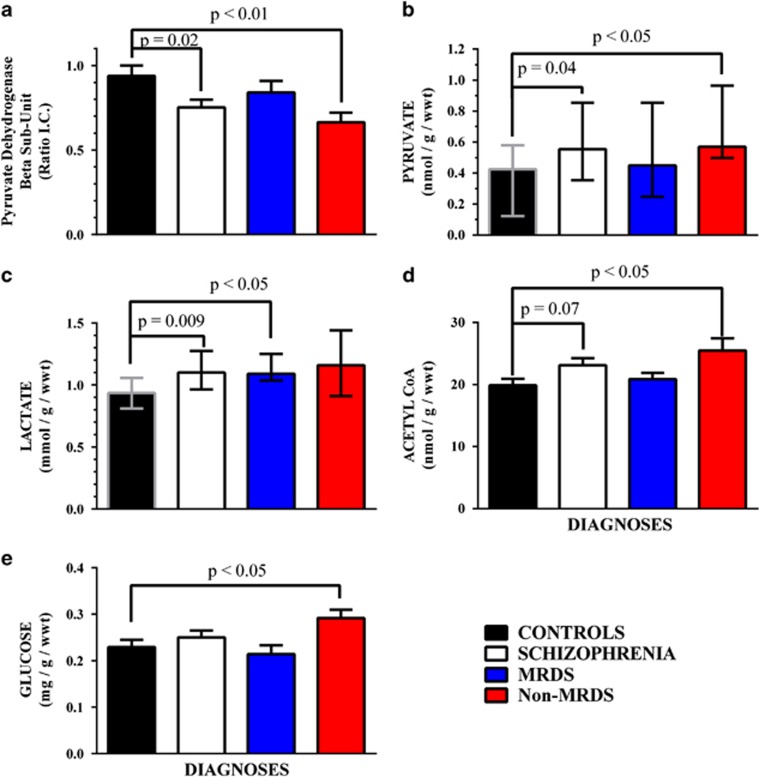
Levels of pyruvate dehydrogenase subunit β (**a**: mean±s.e.m.), pyruvate (**b**: median±IQR), lactate (**c**: median±IQR), acetyl-CoA (**d**: mean±s.e.m.) and glucose (**e**: mean±s.e.m.) in the striatum from controls, subjects with schizophrenia, subjects with schizophrenia and a deficit in cortical muscarinic receptors (MRDS) and subjects with schizophrenia without a deficit in cortical muscarinic receptors (non-MRDS). IQR, interquartile range. MDRS, muscarinic receptor deficit schizophrenia.

Compared with controls, levels of PDHB were lower (−20%) in the striatum from subjects with schizophrenia (Figure 2a). Notably, PDHB levels were lower in the striatum from subjects with in non-MRDS (−29%) but not MRDS. Levels of PDHB did not vary with gender (PDHB Ratio IC mean±s.e.m.: male=0.85±0.07 vs female=0.80±0.07; *P*=0.63) but there was a trend to lower levels of PDHB between suicide completers (0.68±0.10) compared with those who died by other causes (0.85±0.04; *P*=0.07). The regression lines describing the relationships between PDHB and age, CNS pH, PMI, FRADD, LEAD, brain weight, pyruvate, lactate and acetyl-CoA did not deviate significantly from a slope=0 (Table 1).

### Pyruvate

Pyruvate and lactate was measured in the striatum of 20 control subjects and 36 subjects with schizophrenia, which included 18 subjects with MRDS. Demographic and tissue collection data did not vary with diagnoses and rates of suicide, duration of illness or treatment data varied with diagnoses did not vary between MRDS and non-MRDS (Supplementary Table 1B).

Compared with controls, levels of pyruvate were higher (Δ median=31% Δ mean=47%) in the striatum from subjects with schizophrenia (Figure 2b). The higher levels of pyruvate were in the striatum from non-MRDS subjects (Δ median=34% Δ mean=66%), but not in the striatum from subjects with MRDS. Levels of pyruvate did not vary with gender (pyruvate (nmol g^−1^ per wwt) mean±s.e.m.: male=0.74±0.16 vs female=0.95±0.31; *P*=0.56) or between suicide completers (0.88±0.39) compared to those who died by other causes (0.76±0.14; *P*=0.71). In control subjects levels of pyruvate were related to levels of glucose whereas in schizophrenia levels of pyruvate were related to levels of lactate and glucose (Table 1). The regression lines describing the relationships between pyruvate and age, CNS pH, PMI, FRADD, LEAD, and brain weight did not deviate significantly from a slope=0.

### Lactate

Compared with controls, levels of lactate were higher (Δ median=17% Δ mean=15%) in the striatum from subjects with schizophrenia (Figure 2c). Notably, higher levels of lactate appear to be limited to the striatum from subjects with MRDS subjects (Δ median=23% Δ mean=17%), not non-MRDS. Levels of lactate did not vary with gender (lactate (mmol g^−1^ per wwt) mean±s.e.m.: male=1.09±0.05 vs female=1.27±0.08; *P*=0.14) or between suicide completers (1.06±0.05) compared with those who died by other causes (1.15±0.06; *P*=0.40). In controls levels of lactate were related to levels of acetyl-CoA whereas in schizophrenia lactate levels were related to levels of acetyl-CoA and glucose (Table 1). There was a moderate relationship between CNS pH and lactate in both controls and subjects with schizophrenia but the regression line describing the relationships between lactate age, PMI, FRADD, LEAD, brain weight did not deviate significantly from a slope=0 (Table 1).

### Acetyl-CoA

Levels of acetyl-CoA were measured in the striatum of 14 controls and 25 subjects with schizophrenia including 13 subjects with MRDS. There was a trend towards a difference in CNS pH between subjects with schizophrenia and controls (Supplementary Table 1C). There were no significant differences between other demographic, CNS-collection related or treatment variables with diagnoses. There were no significant differences in any demographic, CNS collection or treatment parameters between MRDS, non-MRDS and controls.

Compared with controls, there was a strong trend to higher levels of acetyl-CoA (+14%) in the striatum from subjects with schizophrenia (Figure 2d). Further analysis showed that levels of acetyl-CoA were higher in the striatum of the non-MRDS subjects (+28%) but not in striatum from subjects with MRDS. Acetyl-CoA levels did not vary with gender (acetyl-CoA (nmol g^−1^ per wwt): mean±s.e.m.: male=24±0.19 vs female=22±0.96; *P*=0.30) or between suicide completers (21±1.5) compared with those who died by other causes (21±1.5; *P*=0.42). In schizophrenia, levels of acetyl-CoA were related to levels of glucose (Table 1). The regression lines describing the relationships between acetyl-CoA and age, CNS pH, PMI, FRADD, LEAD and brain weight did not deviate significantly from a slope=0.

### Glucose

Glucose levels were measured in the striatum of 13 controls and 25 subjects with schizophrenia including 14 subjects with MRDS. Compared with controls, there was a trend levels CNS pH being lower for subjects with schizophrenia, but there was no differences in other demographic, CNS-collection related or treatment variables with diagnoses (Supplementary Table 1D). There were no significant differences in any demographic, CNS-collection related or treatment variables differing between subjects with MRDS, non-MRDS or controls.

Levels of glucose did not differ in the striatum from subjects schizophrenia compared with controls (Figure 2e). However, compared with controls, levels of glucose were higher in the striatum of the non-MRDS subjects (+27%) but not in striatum from subjects with MRDS. Levels of striatal glucose did not vary with gender (Glucose (mg g^−1^ per wwt): mean±s.e.m.: male=0.24±0.01 vs female=0.26±0.01; *P*=0.48) or between suicide completers (0.23±0.02) compared with those who died by other causes (0.24±0.01; *P*=0.54). The regression lines describing the relationships between glucose and age, CNS pH, PMI, FRADD, LEAD and brain weight did not deviate significantly from a slope=0 (Table 1).

### Discriminate analyses

**Figure 3:**
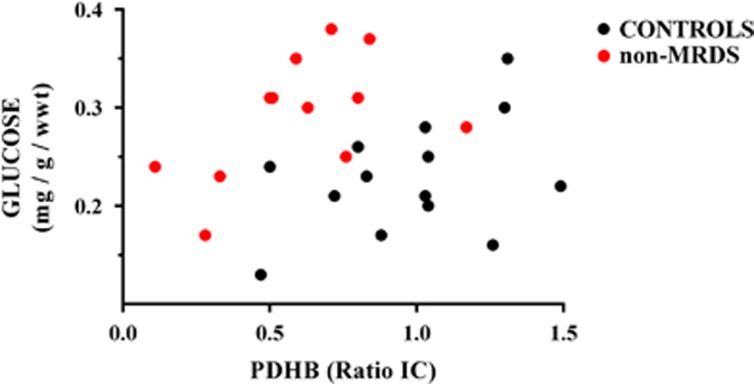
Levels of striatal pyruvate dehydrogenase subunit β (PDHB) and glucose in non-MRDS subjects and controls. IC, internal control; MRDS, muscarinic receptor deficit schizophrenia.

**Figure 4:**
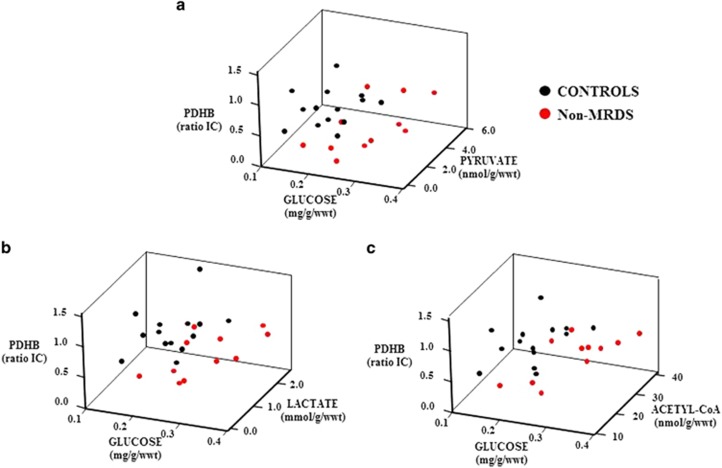
Levels of pyruvate dehydrogenase subunit β (PDHB) compared to levels of pyruvate and glucose (**a**), lactate and glucose (**b**) and acetyl-CoA and glucose (**c**) in the striatum from subjects with non-MRDS and controls. MRDS, muscarinic receptor deficit schizophrenia.

Discriminate analyses of individual analytes did not separate subjects with schizophrenia, MRDS, non-MRDS or controls. By contrast, using striatal levels of PDHB and glucose separated subjects with non-MRDS from controls with a specificity of 93%, selectivity 83% (Figure 3). Using levels of PDHB, pyruvate and glucose (Figure 4a: specificity 93%, selectivity 82%) or PDHB, lactate and glucose (Figure 4b: specificity 93%, selectivity 83%) did not increase the separation between controls and non-MRDS. However, using levels of PDHB, acetyl-CoA and glucose gave a slight improvement in selectivity (Figure 4c: specificity 93%, selectivity 91%). More complex combinations of levels of metabolites did not improve separations between subjects with non-MRDS or controls.

## Discussion

This study presents the data that supports the hypothesis that abnormal striatal glucose metabolism is contributing to the pathophysiology of schizophrenia. This proportion comes from our findings of low levels of PDHB and higher levels of pyruvate, along with a trend to higher levels of acetyl-CoA, in the striatum from subjects with the disorder. These findings would be consistent with decreased activity of the TCA cycle and/or the Embden–Meyer pathway (Figure 1) in subjects with schizophrenia.

Our study design was novel as it was based on the premise that studying the pathophysiology of the syndrome of schizophrenia as a whole may be hindering the identification of the underpinning biochemical bases of individual disorders within the syndrome.^20^ Our data would support that hypothesis because they show that changes glucose, pyruvate, PDHB and acetyl-CoA are limited to subjects with non-MRDS, whereas changes in lactate occur in MRDS. Significantly, using different combinations of measures of glucose metabolisms in discriminate analyses allows the separation of subjects with non-MRDS, but not MRDS, from controls with a high degree of specificity and selectivity. These data argue that changes in striatal glucose metabolism may be a major contributor to the genesis of symptoms associated with subjects with non-MRDS. As changes in markers of glucose metabolism are only detectable in subjects with non-MRDS, but not MRDS who have markedly lower levels of muscarinic receptors in their cortex^33^ and striatum,^24^ it may be significant that some data suggests there is bi-direction links between the activity and/or levels of muscarinic receptors and levels of glucose metabolism.^25, 26^ Hence, our data may indicate that low levels of CNS muscarinic receptors may be protective against changes in glucose metabolism in the subset of subjects with MRDS.

Glucose metabolism is a source of energy for all cells in the CNS but neurons are particularly dependent on glucose as they have little or no glycogen stores that can be mobilized as an alternate source of energy.^40^ Moreover, it has been recently suggested that neuronal activity may be a highly glucose dependent.^41^ Our data using homogenates of human striatum does not allow any conclusions to be proposed on which cells may have increased levels of glucose in subjects with non-MRDS. It will therefore be important to determine which cells in the striatum have decreased levels of PDHB as low levels of the enzyme would appear to be an important measurable marker that could be used in immunohistochemistry studies to identify which cells may have altered levels of glucose metabolism in subjects with non-MRDS. In addition, in using cell homogenates we cannot distinguish whether the high levels of striatal glucose in non-MRDS are intra- or extracellular. If the high levels of glucose are in the extracellular milieu this would suggest there is an inability of striatal cells to take up glucose which would be consistent with neuroimaging studies in schizophrenia.^9, 10, 11, 12, 13, 14^ Glucose uptake by the cells in the striatum is dependent on a number of glucose transporters and therefore determining whether there is a deficiency in glucose transporters in striatum from subjects with non-MRDS may shed light on whether these subjects could have lost the ability to internalize glucose into cells.

As in any study using subjects with schizophrenia treated with antipsychotic drugs, it is possible that changes in glucose metabolism may have resulted from such treatments. Against this argument is the finding that subjects with MRDS and non-MRDS appear to have had similar antipsychotic drug treatment regimes.^33^ This would suggest the change in glucose metabolism in one of these two groups is not likely to be simply an effect of antipsychotic drug treatment. Moreover, subjects with schizophrenia in this study were treated with typical antipsychotic drugs. It is therefore significant that it has been shown that treating rats with chlorpromazine suppresses glycolysis in the CNS, but not the periphery.^42^ In addition, it has been shown that treatment with haloperidol lowered whole brain glucose and pyruvate, caused no change in levels of acetyl-CoA^43^ and increased levels of brain lactate.^16, 43^ Finally, it has been reported that haloperidol did not change striatal metabolic rates measured using [18F]fluorodeoxyglucose in haloperidol-responsive patients with schizophrenia.^44^ These data argue that treatment with typical antipsychotic drugs *per se* do not alter CNS glucose metabolism.

A limitation of our studies is that, owing to limited tissue availability, not all markers could be measured in every case meaning cohort sizes become relatively small. That been said, our smallest cohort sizes are equal to those in many other postmortem CNS studies. In addition, the findings on lactate need to be treated with caution as levels of that metabolite were related to measures of CNS pH. As pH is a measure of CNS tissue preservation^32^ we cannot rule out that the higher levels of lactate in the striatum from subjects with schizophrenia is a postmortem artefact, particularly in the light of reports that lactate in human blood^45^ and cerebellum^16^ increase after death and these increases can be related to falling pH. It has been suggested that CNS glucose increases with PMI in mouse CNS,^46^ which is in marked contrast to our findings where there was no relationship between levels of glucose in the striatum from subjects with schizophrenia (*r*^2^=0.010, *P*=0.74) or controls (*r*^2^=0.013, *P*=0.59) and PMI. This difference may be due to changes after death in mice CNS not modeling changes in human CNS due to the different sizes of the CNS between the two species. Using rat, a rapid rise of CNS acetyl-CoA has been reported minutes after death after which the levels of CNS acetyl-CoA appear to stabilize.^47^ Although these data could suggest that the higher levels of acetyl-CoA we report in the striatum from non-MRDS could be a postmortem artefact, for that to occur levels of acetyl-CoA must have increased to a greater degree in a subset of subjects with schizophrenia (non-MRDS) compared with another subset of subjects with schizophrenia (MRDS) and controls. It is difficult to provide a convincing hypothesis as to how this selected change in acetyl-CoA could have occurred. Hence, other than the measure of lactate, our data would suggest that the changes we report in striatum from subjects with non-MRDS are unlikely to be solely due to some postmortem artefact.

We have previously reported that subjects with MRDS have been shown to have a widespread loss of cortical muscarinic receptors,^21, 22^ have disruptions in muscarinic M1 receptor signaling,^48^ have a distinct pattern of muscarinic M1 receptor gene promoter methylation and higher levels of a microRNA that acts to reduce receptor expression,^49^ and have a reduced response to a muscarinic M1-receptor-positive allosteric modulator.^50^ In addition, we have reported that subjects with MRDS also have decreased radioligand binding to striatal muscarinic receptors and higher levels of post-synaptic density protein 95,^24^ showing changes in the molecular cytoarchitecture of the CNS in subjects with MRDS extends beyond the cortex. Thus, to our knowledge, this study is different in that we have shown the majority of changes in markers of glucose metabolism are present in the striatum from subjects with non-MRDS.

In conclusion, we have shown a change in levels of a number of markers in the striatum of subjects with schizophrenia that are consistent with changes in glucose metabolism, and that these changes are restricted to subjects with non-MRDS. These findings reinforce the argument that studying subgroups of subjects within the syndrome of schizophrenia does lead to being able to better understand the pathophysiology of the different disorders in the syndrome. These systematic changes allow the differentiation of subjects with non-MRDS from controls with a high degree of selectivity and specificity suggesting changes in glucose metabolism is an important component of the pathophysiology of non-MRDS but not MRDS. Recently, there has been a major focus on potential abnormalities in peripheral glucose metabolism in subjects with schizophrenia that are either caused or worsened by treatment with certain antipsychotic drugs.^51^ Our data would suggest that a continued focus on changes in glucose metabolism in the CNS from subjects with schizophrenia will also be important in better understanding the pathophysiology of the disorder and whether treatment with the antipsychotic drugs peripheral glucose homeostasis may be detrimental to CNS homeostasis.

## Figures and Tables

**Table 1:** Relationships levels of different analytes in human striatum and the same analytes and demographic, CNS collection and pharmacological data from the cases from whom the striatum was collected

	n		*Age*	*pH*	*PMI*	*DI*	*FRADD*	*LEAD*	*Brain weight*	*Pyruvate*	*Lactate*	*Acetyl-CoA*	*Glucose*
*PDHB*													
Controls	20	*r*^2^	<0.001	0.095	0.094				0.011	0.023	0.127	0.166	0.075
		*P*	0.94	0.19	0.19				0.66	0.52	0.12	0.15	0.37
Schizophrenia	40	*r*^2^	0.042	0.016	0.017	0.021	0.003	0.036	0.021	0.003	<0.001	0.015	0.048
		*P*	0.21	0.45	0.43	0.37	0.76	0.34	0.42	0.77	0.86	0.55	0.29
													
*Pyruvate*													
Controls	20	*r*^2^ (*ρ*)	0.023	0.067	0.014				0.094		(0.260)	(0.175)	**(0.520)**
		*P*	0.52	0.27	0.62				0.20		0.28	0.43	**0.010**
Schizophrenia	36	*r*^2^ (*ρ*)	0.270	0.076	0.038	0.003	0.030	0.115	0.084		**(0.370)**	(0.175)	**(0.521)**
		*P*	0.33	0.10	0.26	0.72	0.31	0.10	0.11		**0.02**	0.42	**0.01**
													
*Lactate*													
Controls	20	*r*^2^ (*ρ*)	<0.001	**0.290**	0.006				0.009			**(0.760)**	(0.007)
		*P*	0.74	**0.01**	0.75				0.70			**0.002**	0.98
Schizophrenia	36	*r*^2^ (*ρ*)	<0.001	**0.262**	0.018	<0.001	< 0.001	0.074	0.059			**(0.630)**	**(0.670)**
		*P*	0.91	**0.001**	0.44	0.87	0.89	0.19	0.18			**0.001**	**<0.001**
													
*Acetyl-CoA*													
Controls	14	*r*^2^	0.012	0.24	< 0.001				0.084				0.129
		*P*	0.71	0.07	0.97				0.31				0.23
Schizophrenia	25	*r*^2^	0.044	0.072	0.002	0.007	0.002	0.013	0.009				**0.258**
		*P*	0.31	0.20	0.83	0.68	0.85	0.59	0.68				**0.02**
													
*Glucose*													
Controls	13	*r*^2^	0.609	0.102	0.010				0.083				
		*P*	0.002	0.29	0.74				0.34				
Schizophrenia	14	*r*^2^	0.020	0.094	0.013	0.066	0.063	< 0.001	0.096				
		*P*	0.50	0.14	0.59	0.22	0.31	0.96	0.17				

Abbreviations: CNS, central nervous system; DI, duration of illness; FRADD, final recorded drug dose expressed as chlorpromazine equivalents per day; LEAD, lifetime exposure to antipsychotic drugs expressed as chlorpromazine equivalents per year × 10^−3^; PDHB, pyruvate dehydrogenase; PMI, postmortem interval. Relationships where the linear regression line deviated significantly from zero are shown in bold.
