# Supplementary material for: Evidence for impaired glucose metabolism in the striatum, obtained postmortem, from some subjects with schizophrenia
Source: Transl Psychiatry. 2016 Nov 15;6(11):e949–. doi: 10.1038/tp.2016.226 (PMC5314134; doi:10.1038/tp.2016.226)
Supplement: Supplementary Table 1 [file tp2016226x1.docx]

Supplementary Table 1: Summaries of the demographic, treatment and pharmacological data for cases used in different aspects of the study of pyruvate dehydrogenase β sub-unit, pyruvate, lactate, acetyl-CoA and glucose in the striatum.

| **A** | **PDHB** |  |  |  |  |  |  |  |  |  |
| --- | --- | --- | --- | --- | --- | --- | --- | --- | --- | --- |
|  |  | Sex (M/F) | Age (yr) | Sui (Y/N) | CNS pH | PMI (hr) | Brain Weight (gm) | DI (yr) | FRADD | LEAD |
|  | Controls | 16 / 4 | 46 ± 3.8 | 0 / 20 | 6.32 ± 0.05 | 44 ± 3.6 | 1388 ± 31 |  |  |  |
|  | Schizophrenia | 31 / 8 | 47 ± 2.6 | 13 / 26 | 6.24 ± 0.03 | 40 ± 2.1 | 1401 ± 34 | 21 ± 2.5 | 658 ± 111 | 11 ± 2.5 |
|  | p | 1.00 | 0.73 |  | 0.13 | 0.41 | 0.79 |  |  |  |
|  |  |  |  |  |  |  |  |  |  |  |
|  | MRDS | 16 / 4 | 47 ± 3.8 | 7 / 13 | 6.25 ± 0.05 | 39 ± 2.7 | 1432 ± 35 | 20 ± 3.5 | 598 ± 154 | 13 ± 3.9 |
|  | non-MRDS | 15 / 4 | 48 ± 3.7 | 6 / 13 | 6.22 ± 0.05 | 41 ± 3.2 | 1368 ± 59 | 22 ± 3.5 | 732 ± 164 | 9.9 ± 3.1 |
|  | F |  | 0.12 |  | 1.27 | 0.49 | 0.59 |  |  |  |
|  | d.f. |  | 2,56 |  | 2,56 | 2,56 | 2,49 |  |  |  |
|  | p | 1.00 | 0.89 | 1.00 | 0.29 | 0.62 | 0.56 | 0.7 | 0.56 | 0.6 |
|  |  |  |  |  |  |  |  |  |  |  |
| **B** | **PYRUVATE AND LACTATE** | |  |  |  |  |  |  |  |  |
|  | Controls | 16 / 4 | 46 ± 3.8 | 0 / 20 | 6.32 ± 0.05 | 44 ± 3.6 | 1388 ± 31 |  |  |  |
|  | Schizophrenia | 28 / 8 | 46 ± 2.7 | 13 / 23 | 6.23 ± 0.03 | 40 ± 2.2 | 1402 ± 35 | 20 ± 2.5 | 656 ± 115 | 11 ± 2.6 |
|  | p | 1.00 | 0.95 |  | 0.1 | 0.43 | 0.79 |  |  |  |
|  |  |  |  |  |  |  |  |  |  |  |
|  | MRDS | 14 / 4 | 45 ± 4.0 | 7 / 11 | 6.22 ± 0.04 | 39 ± 3.0 | 1444 ± 35 | 18 ± 3.6 | 586 ± 166 | 12 ± 4.2 |
|  | non-MRDS | 14 / 4 | 47 ± 3.9 | 6 / 12 | 6.24 ± 0.05 | 41 ± 3.3 | 1357 ± 62 | 22 ± 3.7 | 732 ± 164 | 10 ± 3.3 |
|  | F |  | 0.07 |  | 1.43 | 0.40 | 0.99 |  |  |  |
|  | d.f. |  | 2,53 |  | 2,53 | 2,54 | 2,47 |  |  |  |
|  | p | 0.98 | 0.93 | 0.74 | 0.25 | 0.67 | 0.38 | 0.47 | 0.54 | 0.76 |
|  |  |  |  |  |  |  |  |  |  |  |
| **C** | **ACETYL-CoA** |  |  |  |  |  |  |  |  |  |
|  | Controls | 12 / 2 | 48 ± 4.4 | 0 / 14 | 6.36 ± 0.05 | 44 ± 4.8 | 1404 ± 38 |  |  |  |
|  | Schizophrenia | 20 / 5 | 48 ± 3.2 | 9 / 16 | 6.24 ± 0.04 | 42 ± 2.8 | 1394 ± 44 | 20 ± 2.9 | 613 ± 140 | 12 ± 3.6 |
|  | p | 1.00 | 0.91 |  | 0.05 | 0.75 | 0.87 |  |  |  |
|  |  |  |  |  |  |  |  |  |  |  |
|  | MRDS | 10 / 3 | 43 ± 4.4 | 5 / 8 | 6.23 ± 0.04 | 40 ± 3.7 | 1428 ± 51 | 16 ± 3.8 | 638 ± 235 | 14 ± 5.7 |
|  | non-MRDS | 10 / 2 | 52 ± 4.7 | 4 / 8 | 6.25 ± 0.06 | 45 ± 4.3 | 1356 ± 75 | 24 ± 24.1 | 583 ± 154 | 11 ± 24.5 |
|  | F |  | 1.20 |  | 2.10 | 0.31 | 0.42 |  |  |  |
|  | d.f. |  | 2,36 |  | 2,36 | 2,38 | 2,32 |  |  |  |
|  | p | 0.92 | 0.32 | 1.00 | 0.14 | 0.73 | 0.66 | 0.13 | 0.85 | 0.73 |
|  |  |  |  |  |  |  |  |  |  |  |
| **D** | **GLUCOSE** |  |  |  |  |  |  |  |  |  |
|  |  |  | Age (yr) |  | CNS pH | PMI (hr) | Brain Weight (gm) | DI (yr) | FRADD | LEAP |
|  | Controls | 11 / 2 | 48 ± 4.8 | 0 / 13 | 6.36 ± 0.05 | 42 ± 4.9 | 1425 ± 33 |  |  |  |
|  | Schizophrenia | 21 / 4 | 48 ± 3.3 | 9 / 16 | 6.24 ± 0.04 | 41 ± 2.9 | 1437 ± 38 | 19 ± 2.8 | 658 ± 154 | 12 ± 3.5 |
|  | p | 1.00 | 0.99 |  | 0.06 | 0.80 | 0.83 |  |  |  |
|  |  |  |  |  |  |  |  |  |  |  |
|  | MRDS | 12 / 2 | 47 ± 4.0 | 5 / 9 | 6.24 ± 0.04 | 38 ± 3.5 | 1451 ± 46 | 18 ± 3.7 | 583 ± 217 | 13 ± 5.3 |
|  | non-MRDS | 9 / 2 | 50 ± 5.6 | 4 / 7 | 6.23 ± 0.06 | 45 ± 4.6 | 1419 ± 67 | 21 ± 4.4 | 752 ± 226 | 11 ± 4.7 |
|  | F |  | 0.08 |  | 1.87 | 0.70 | 0.12 |  |  |  |
|  | d.f. |  | 2,35 |  | 2,35 | 2,35 | 2,31 |  |  |  |
|  | p | 0.96 | 0.93 | 1.00 | 0.16 | 0.50 | 0.88 | 0.62 | 0.60 | 0.78 |

Abbreviations: DI = duration of illness, F = female, FRADD = final recorded drug dose expressed as chlorpromazine equivalents per day, LEAD = Lifetime Exposure to Antipsychotic Drugs expressed as chlorpromazine equivalents per year x 10^-3^, M = male, PMI = postmortem interval, Sui = suicide completers.
