# Supplementary material for: Evidence for impaired glucose metabolism in the striatum, obtained postmortem, from some subjects with schizophrenia
Source: Transl Psychiatry. 2016 Nov 15;6(11):e949–. doi: 10.1038/tp.2016.226 (PMC5314134; doi:10.1038/tp.2016.226)
Supplement: Supplementary Table 2 [file tp2016226x2.pdf]

Supplementary Table 2: Demographic, pharmacological and tissue collection data for tissue donors.

| | Sex | Age (yr) | Suicide | Cause of Death | pH | PMI (hr) | DI (yr) | Antipsychotic drug | FRADD | LEAP | Brain Weight (gms) |
|---|---|---|---|---|---|---|---|---|---|---|---|
| **Controls** | M | 36.6 | | Crush Accident | 6.46 | 42 | | | | | 1350 |
| | F | 68.5 | | Asthma | 6.32 | 38 | | | | | 1340 |
| | M | 50.2 | | Ischaemic Heart Disease | 6.40 | 65 | | | | | 1130 |
| | M | 24.8 | | Exsanguination | 6.48 | 50 | | | | | 1266 |
| | M | 53.8 | | Ischaemic Heart Disease | 6.56 | 44.5 | | | | | 1515 |
| | M | 22.6 | | Exsanguination | 6.58 | 51 | | | | | 1370 |
| | M | 42.0 | | Cardiomegally | 6.34 | 63 | | | | | 1385 |
| | F | 66.2 | | Acute Myocardial Infarction | 6.37 | 43 | | | | | 1454 |
| | F | 21.0 | | Myocarditis | 6.03 | 58 | | | | | 1200 |
| | M | 21.4 | | Acute Epiglottitis | 5.82 | 40 | | | | | |
| | M | 25.4 | | Electrocution | 6.42 | 24 | | | | | 1370 |
| | M | 48.9 | | Coronary Atheroma | 6.37 | 24 | | | | | 1295 |
| | M | 72.5 | | Coronary Atheroma | 6.21 | 39 | | | | | 1182 |
| | M | 68.7 | | Aortic Stenosis | 6.06 | 41 | | | | | 1530 |
| | M | 64.5 | | Coronary Atheroma | 6.59 | 69 | | | | | 1430 |
| | M | 52.4 | | Pulmonary Embolism | 5.98 | 22 | | | | | 1360 |
| | M | 44.0 | | Coronary Atheroma | 6.43 | 51 | | | | | 1530 |
| | M | 52.1 | | Pulmonary Thromboembolism | 6.34 | 12 | | | | | 1590 |
| | F | 39.2 | | Mitral valave prolapse | 6.38 | 65 | | | | | 1595 |
| | M | 42.3 | | Coronary Atheroma | 6.32 | 26 | | | | | 1480 |
| **MRDS** | M | 36.5 | Y | Drug overdose | 6.04 | 38 | 12 | Fluphenazine | 200 | 2.40 | 1600 |
| | M | 51.5 | N | Ischaemic Heart Disease | 5.98 | 20 | 32 | Fluphenazine<br>Thioridazine | 2000 | 64.00 | 1585 |
| | F | 21.8 | Y | Carbon Monoxide Poisoning | 6.24 | 56 | 2 | Haloperidol | | | 1310 |
| | M | 26.0 | Y | Combined Drug Toxicity | 6.38 | 49 | 2 | Trifluoperazine | 200 | 0.40 | 1595 |
| | M | 53.4 | N | Intestinal Ischaemia | 5.98 | 37 | 30 | Fluphenazine<br>Chlorpromazine | 1700 | 51.00 | 1421 |
| | M | 67.5 | N | Pneumonia | 6.46 | 21 | 36 | Fluphenazine | 75 | 2.70 | 1564 |

| | | | | | | | | | | | |
|---|---|---|---|---|---|---|---|---|---|---|---|
| | M | 71.2 | N | Aspiration Food Bolus | 6.45 | 48 | 53 | Thioridazine | 150 | 7.95 | 1500 |
| | M | 53.2 | N | Aspiration Food Bolus | 6.23 | 43 | 7 | Off Drug | | 1.40 | 1445 |
| | M | 69.9 | N | Ischaemic Heart Disease | 6.38 | 44.5 | 47 | Trifluoperazine | 100 | 4.70 | 1250 |
| | F | 68.2 | N | Ischaemic Heart Disease | 5.73 | 42 | 40 | Trifluoperazine | 400 | 16.00 | 1300 |
| | M | 22.9 | Y | Combined Drug Toxicity | 6.17 | 37 | 3 | Pimozide | 200 | 0.60 | 1312 |
| | F | 65.6 | N | Rupture Abdominal Aneurysm | 6.35 | 50 | 18 | Fluphenazine<br>Haloperidol | 550 | 9.90 | |
| | M | 41.3 | Y | Combined Drug Toxicity | 6.20 | 31 | 11 | Fluphenazine<br>Trifluoperazine | 500 | 5.50 | |
| | M | 53.1 | N | Coronary Atheroma | 6.29 | 9 | 9 | Trifluoperazine<br>Chlorpomazine | 300 | 2.70 | 1420 |
| | M | 19.0 | Y | Unascertained | 6.22 | 43 | 3 | Haloperidol | 750 | 2.25 | 1405 |
| | M | 42.9 | N | Coronary Atheroma | 6.26 | 47 | 22 | Off Drug | | 22.00 | 1440 |
| | M | 26.8 | Y | Carbon Monoxide Poisoning | 6.39 | 52 | 2 | Off Drug | | 1.00 | 1655 |
| | F | 47.3 | N | Pneumonia | 6.31 | 50 | 20 | Off Drug (Thioridazine) | | 12.00 | 1120 |
| | M | 48.8 | N | Bronchopneumonia | 6.62 | 30 | 24 | Flupenthixol<br>Thioridazine | 1250 | 30.00 | |
| | M | 44.4 | N | Ischaemic Heart Disease | 6.28 | 32 | 23 | Thioridazine | 600 | 13.80 | 1425 |
| **Non-MRDS** | F | 72.1 | N | COAD | 5.84 | 36 | 48 | | | 0.00 | 1178 |
| | M | 66.8 | N | Bronchopneumonia | 6.49 | 39.5 | 45 | Chlorpromazine<br>Haloperidol | 1200 | 54.00 | 1675 |
| | M | 22.0 | Y | Carbon Monoxide Poisoning | 6.06 | 41.5 | 4 | Haloperidol decanoate<br>Haloperidol | 1900 | 7.60 | 1375 |
| | M | 47.3 | N | Ischaemic Heart Disease | 6.41 | 32.5 | 27 | Fluphenazine decanoate<br>Thioridazine | 530 | 14.31 | 1475 |
| | M | 27.9 | Y | Burning | 6.28 | 22 | 8 | Chlorpromazine<br>Pimozide | 1200 | 9.60 | 1575 |
| | F | 72.4 | N | Pneumonia | 6.48 | 58.5 | 37 | Chlorpromazine | 25 | 0.93 | 1554 |
| | M | 48.7 | Y | Multiple Injuries | 6.52 | 41.5 | 21 | Chlorpromazine<br>Haloperidol decanoate | 1400 | 29.40 | |

| | | | | | | | | | | |
|---|---|---|---|---|---|---|---|---|---|---|
| M | 22.7 | N | Pericarditis | 6.07 | 37 | 3 | Off Drug | | 1.35 | 1595 |
| M | 38.1 | N | Meningo Encephalitis | 6.02 | 50 | 4 | Off Drug | | 0.40 | 1025 |
| F | 35.4 | N | Coronary Artery Thrombosis | 6.26 | 15 | 7 | Haloperidol | 300 | 2.10 | 1680 |
| M | 55.2 | N | Coronary Atheroma | 6.10 | 25 | 33 | Thioridazine | 400 | 13.20 | |
| F | 48.3 | N | Pulmonary Thromboembolism | 6.21 | 52.5 | 22 | Fluphenazine decanoate | 700 | 15.40 | 1305 |
| | | | | | | | Chlopromazine | | | |
| M | 65.0 | N | Bronchopneumonia | 6.29 | 42 | 36 | Trifluoperazine | 460 | 16.56 | 1505 |
| | | | | | | | Haloperidol decanoate | | | |
| M | 53.0 | N | Cancer breast | 6.17 | 42 | 11 | Off Drug | | | 1200 |
| M | 38.9 | N | Mediastinitis | 5.52 | 40 | 15 | Haloperidol decanoate | 160 | 2.40 | |
| M | 42.3 | Y | Hanging | 6.44 | 47 | 8 | Haloperidol decanoate | 128 | 1.02 | |
| M | 23.6 | Y | Multiple Injuries | 6.19 | 78 | 5 | Off Drug | | 1.50 | 1050 |
| M | 70.1 | N | Bronchopneumonia | 5.80 | 46 | 20 | | | | 1530 |
| M | 54.0 | Y | Hanging | 6.49 | 35.5 | 38 | Olanzapine | | | 1110 |
| M | 54.8 | N | Pneumonia | 6.08 | 46.8 | 36 | Flupenthixol | 550 | 19.80 | 1060 |
